# Feasibility of automated proton therapy plan adaptation for head and neck tumors using cone beam CT images

**DOI:** 10.1186/s13014-016-0641-7

**Published:** 2016-04-30

**Authors:** Christopher Kurz, Reinoud Nijhuis, Michael Reiner, Ute Ganswindt, Christian Thieke, Claus Belka, Katia Parodi, Guillaume Landry

**Affiliations:** Department of Radiation Oncology, LMU Munich, Munich, Germany; Department of Medical Physics, Faculty of Physics, Ludwig-Maximilians-Universität München, Munich, Germany

**Keywords:** Intensity modulated proton therapy, Cone beam CT, Adaptive radiation therapy, Deformable image registration, Head and neck cancer

## Abstract

**Background:**

Intensity modulated proton therapy (IMPT) of head and neck (H&N) tumors may benefit from plan adaptation to correct for the dose perturbations caused by weight loss and tumor volume changes observed in these patients. As cone beam CT (CBCT) is increasingly considered in proton therapy, it may be possible to use available CBCT images following intensity correction for plan adaptation. This is the first study exploring IMPT plan adaptation on CBCT images corrected and delineated by deformable image registration of the planning CT (pCT) to the CBCT, yielding a virtual CT (vCT).

**Methods:**

A Morphons algorithm was used to deform the pCTs and corresponding delineations of 9 H&N cancer patients to a weekly CBCT acquired within ±3 days of a control replanning CT scan (rpCT). The IMPT treatment plans were adapted using the vCT and the adapted and original plans were recalculated on the rpCT for dose/volume parameter evaluation of the impact of adaptation.

**Results:**

On the rpCT, the adapted plans were equivalent to the original plans in terms of target volumes *D*_95_ and *V*_95_, but showed a significant reduction of *D*_2_ in these volumes. OAR doses were mostly equivalent or reduced. In particular, the adapted plans did not reduce parotid gland *D*_mean_, but the dose to the optical system. For three patients the spinal cord or brain stem received higher, though well below tolerance, maximum dose. Subsequent tightening of the treatment planning constraints for these OARs on new vCT-adapted plans did not degrade target coverage and yielded pCT equivalent plans on the vCT.

**Conclusions:**

An offline automated procedure to generate an adapted IMPT plan on CBCT images was developed and investigated. When evaluating the adapted plan on a control rpCT we observed reduced *D*_*2*_ in target volumes as major improvement. OAR sparing was only partially improved by the procedure. Despite potential limitations in the accuracy of the vCT approach, an improved quality of the adapted plans could be achieved.

**Electronic supplementary material:**

The online version of this article (doi:10.1186/s13014-016-0641-7) contains supplementary material, which is available to authorized users.

## Background

Intensity modulated radiation therapy (IMRT) with photons can be considered current state-of-the-art for the treatment of head and neck (H&N) cancer [1–3]. However, intensity modulated proton therapy (IMPT) using spot scanning has been shown to yield superior dose distributions compared to IMRT for the treatment of H&N cancer patients [4–8]. Still, plan adaptation may be desirable to optimally treat patients undergoing weight loss or other anatomical modifications during the radiation course [9–11]. To enable adaptive radiation therapy (ART), a time point for re-planning needs to be chosen and a new delineated diagnostic quality computed tomography (CT) image of the patient is required. The time point may be fixed for all patients based on clinical experience, or a patient specific action level may be derived based on e.g., evaluation of the dose of the day. It is thus obvious that access to frequent, delineated diagnostic CT quality images is of high importance in an adaptive workflow.

In-room cone beam CT (CBCT) imaging, which is increasingly considered for proton therapy [12], provides information related to patient positioning as well as anatomical variations. However, direct dose calculation on CBCT images is not recommended for IMPT [13] due to their poor image quality [14, 15]. Previous work [16–21] suggests that it is feasible to apply deformable image registration (DIR) to the original delineated planning CT (pCT) image to match the anatomy observed on CBCT images. This method of CBCT intensity correction yields an image, referred to as virtual CT (vCT) in this work, suitable to IMPT dose calculation [22–26] and automatically yields updated contours from the DIR [18, 23, 27]. A previous evaluation in terms of matching features and Dice coefficient showed good agreement between vCT contours and those from a physician [23].

The availability of a delineated CT image fulfills the basic technical requirements for adaptation of the IMPT plan. Furthermore, the automated nature of the vCT generation and delineation may allow for plan adaptation with little increase to the clinical workload. To the best of our knowledge there is no study in the literature reporting on plan adaptation for IMPT based on CBCT images. In this study we investigated the re-optimization of the initial IMPT plan using the automatically delineated vCT to restore the initial optimization objectives. We investigated the impact of this correction by recalculating the dose distributions from the original and updated plans on a delineated control replanning CT (rpCT) image taken within 3 days of the CBCT image used to generate the vCT by evaluating dose/volume histogram (DVH) parameters.

## Methods

Clinical datasets of 9 H&N cancer patients undergoing IMRT were employed in this study. The patient data acquisition and anonymization protocol used in this study received approval from the ethics committee of the University Clinic of the Ludwig-Maximilians-Universität München (LMU Munich). 6 patients had lesions of the larynx or pharynx (Pat1-6) while 3 cases had nasal cavity lesions (Pat7-9) as detailed in Table 1. Dose prescriptions in this study follow the original clinical IMRT treatments. The sizes of the target volumes can be found in Additional file 1: Table S1. Each patient had a delineated pCT, a CBCT and delineated rpCT from the same physician. The pCT and rpCT images were acquired with a Toshiba Aquilion LB scanner and reconstructed on a 1.074 mm × 1.074 mm × 3 mm grid. The CBCT images were acquired with the on-board Elekta Synergy Linac imager equipped with XVI R4.5 and reconstructed on a 1 mm × 1 mm × 1 mm grid. The scan parameters were the same as in Landry et al. [23] IMPT plans were generated with a research version of a commercial treatment planning system (TPS) (RayStation 4.6, RaySearch Laboratories, Stockholm, Sweden) following the approach outlined in Kurz et al. [13]: A four field arrangement has been used for Pat1-6 with larynx or pharynx lesions (45°, 90°, 270° and 315° on the International Electrotechnical Commission (IEC) scale, with 90° and 270° blocked in the shoulder area), and a 3 field arrangement for Pat7-9 with nasal cavity lesions (0°, 100°, 260° on the IEC scale, with 0° blocked in the nasal/buccal area). For eight patients simultaneous integrated boost (SIB) plans using two dose levels (reported in Table 1) were generated, while for one patient only a single dose level was used, following again the dose prescriptions of the original clinical IMRT plans. Target definition was also adopted from clinical IMRT planning procedures. The high dose CTV has been retrieved from expansion of the GTV by a 5–7 mm margin, for high dose PTV generation an additional 5–7 mm margin was applied. The low dose CTV also covered the lymph node areas following the delineation approach of Grégoire et al. [28] It was extended by 5–7 mm to the low dose PTV. All treatment fields have been optimized simultaneously (multi-field optimization). For Pat1-6, the main PTV coverage limitation was the mean dose to the parotid glands, since the spinal cord and brain stem were easily spared by IMPT. For Pat7-9 the mean dose to the eye lens and the maximum dose to the optical nerves and chiasm were also critical. Planning risk volumes (PRV) were used for optimization for the spinal cord, brain stem, optical nerves and chiasm. Table 2 reports the TPS-specific DVH objectives employed for treatment plan optimization in this study. Treatment plans were accepted when exceeding a *V*_*95*_ of 95 % in the PTVs, except from two cases where a slightly lower *V*_*95*_ (94 %) was accepted due to OAR constraints.

**Table 1:** Patient characteristics. Δt_rpCT_ and Δt_CBCT_ are the times between planning and replanning CT acquisition and between planning CT and CBCT acquisition respectively

Patient identifier	Age	Sex	Tumor site	TNM stage	Δt_rpCT_ (days)	Δt_CBCT_ (days)	SIB prescription low dose/high dose (Gy)	Number of SIB fractions
Pat1	65	M	Larynx	pT2pN0M0	51	50	50/-	25
Pat2	54	F	Hypopharynx, esophagus	cT4cN2M0	39	41	50.4/56	28
Pat3	71	M	Larynx	pT1bN0M0	34	35	54/60	30
Pat4	87	M	Hypopharynx	cT2cN2bM0	33	34	54/60	30
Pat5	49	M	Nasopharynx	cT2cN2bM0	40	40	54/60	30
Pat6	42	M	Larynx	pT2bpN1M0	44	41	50.4/56	28
Pat7	66	M	Right paranasal sinus	pT2cN0M0	31	30	50.4/61.6	28
Pat8	67	M	Left paranasal sinus	pT3N2bM0	30	30	54.4/64	32
Pat9	76	M	Nasal cavity	cT3N0M0	37	38	50.4/56	28

**Table 2:** DVH parameters used in this study. Planning risk volumes were used for the brain stem, spinal cord, optic nerves and optic chiasm. *D*_prescr._ is the prescription dose. The TPS-specific DVH objectives used in treatment plan optimization are also presented

Organ at risk or target	DVH parameters evaluated	DVH objectives used for planning
High dose PTV	*D*_95_, *D*_2_ *, V*_95_	*V*_95_ = 100 %, *D*_min_ = 100 % · *D*_prescr._, *D*_max_ < 105 % · *D*_prescr._
High dose CTV	*D*_95_, *D*_2_	
Low dose PTV	*D*_95_, *D*_2_ *, V*_95_	*V*_95_ = 100 %, *D*_min_ = 100 % · *D*_prescr._, *D*_max_ < 105 % · *D*_prescr._
Low dose CTV	*D*_95_, *D*_2_	
Parotid glands	*D*_mean_	*D*_mean_ < 26 Gy
Spinal cord	*D*_2_	*D*_max_ < 53 Gy
Brain stem	*D*_2_	*D*_max_ < 53 Gy
Optical nerves	*D*_2_	*D*_max_ < 54 Gy
Optical chiasm	*D*_2_	*D*_max_ < 56 Gy
Eye	*D*_2_	*D*_max_ < 45 Gy
Eye lens	*D*_mean_	*D*_mean_ < 10 Gy

vCT images were generated for each CBCT by performing DIR of the aligned pCT to the CBCT using a Morphons algorithm [29], which is image intensity independent. The tools described in Landry et al. were used with slight modifications to the procedure. In Landry et al. a translational registration was used for aligning the pCT and CBCT prior to DIR [23], while in this work we also allowed for small rotations (always below 5° in this study) to simulate modern 6-degrees-of-freedom patient position correction. The rigid registration was performed on a region of interest (ROI) containing the spine and ranging from the 1^st^ to the 6^th^ vertebrae for Pat1 to Pat6 and on the skull for Pat7 to Pat9. For evaluation, the rpCT was also rigidly registered to the pCT using the same ROIs. Manual tuning of the final registrations yielded corrections smaller than 1 mm, confirming the quality of the rigid registrations. The cavity correction method described in Landry et al., where empty/full cavities on the CBCT take the CT numbers of air/water on the vCT, was used for all cases [23].

The registered rpCT and vCT were imported in the TPS with their respective contours and the dose distribution corresponding to the initial plan, optimized on basis of the pCT, was calculated on the rpCT. A new plan was designed on the vCT using the same optimization parameters as the initial plan and the DIR-based contours. The rpCT dose distribution corresponding to this adapted plan was compared to the one from the original plan using the DVH parameters listed in Table 2 and the corresponding rpCT contours.

The median and interquartile range of the distribution of the differences between the parameters from Table 2 evaluated for the pCT and rpCT (with and without vCT-based plan adaptation) was computed for the 9 patients. DVH parameters with and without plan adaptation for the rpCT were also compared by a paired Wilcoxon signed-rank test in order to find statistically significant differences caused by plan adaptation on basis of the vCT.

## Results

**Fig. 1:**
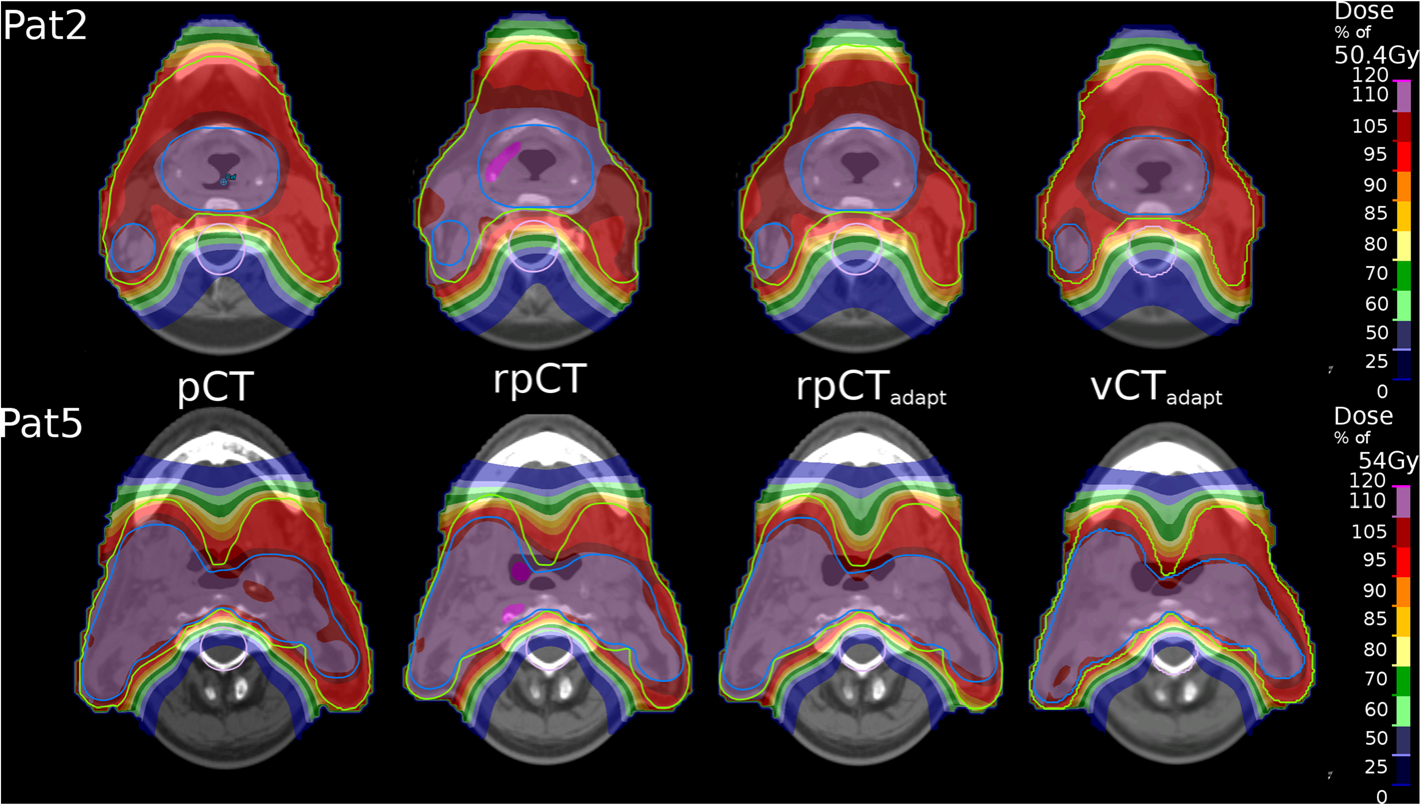
Dose distributions for Pat2 and Pat5 from the original plan on the pCT and rpCT as well as the adapted dose distributions on the rpCT (rpCT_adapt_) and vCT (vCT_adapt_). The high and low dose PTVs are indicated in blue and green respectively. The spinal cord PRV is also shown. For the high dose PTV, the 95 % dose level corresponds to 105 % on the color bar, which is relative to the low dose PTV prescription. Hot spots correspond to 120 % or more of the low dose PTV prescription

Figure 1 shows IMPT dose distributions from the original plan computed on the pCT and rpCT as well as the vCT-adapted plan on the rpCT (labelled rpCT_adapt_) and on the vCT (labelled vCT_adapt_) for two representative patients. Over-dosed regions in the low dose PTV were observed on the rpCT in the vicinity of the high dose PTV and the skin when applying the original plan for Pat2. This over-dosage was reduced by adapting the plan using the vCT. For Pat5 over-dosage was observed (up to 109 % of the high dose PTV prescription dose, which corresponds to about 120 % of the low dose PTV prescription dose) in the high dose PTV and could be eliminated in the adapted plan. In both cases, dose distributions of the adapted plan on the vCT were found similar to the original planning scenario (pCT).

**Fig. 2:**
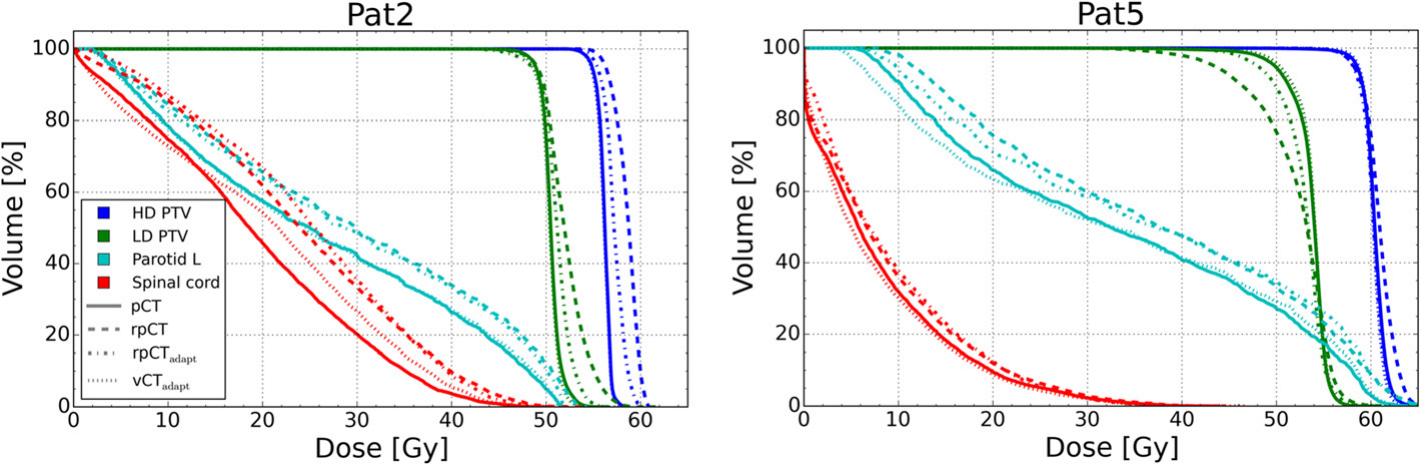
DVH curves for Pat2 and Pat5 for the high dose PTV (HD PTV), low dose PTV (LD PTV), left (L) parotid gland and spinal cord PRV for the dose distributions of the initial plan on the pCT, rpCT and the adapted plan on the rpCT (rpCT_adapt_) and vCT (vCT_adapt_). The LD PTV excludes the HD PTV volume expanded by a 5 mm margin

Figure 2 presents DVH curves for the original plan on the pCT and rpCT as well as the adapted plan on the rpCT and the vCT for the same two patients presented in Fig. 1. For both patients, over-dosed regions were observed in the low dose and high dose PTVs for the original plan on the rpCT. For Pat5, the target coverage of the original plan for the low dose PTV was degraded on the rpCT (*D*_95_ reduction of 4 Gy); for the adapted plan, coverage was almost recovered. The adapted plans mitigated over-dosage (above 105 % of prescription) for both targets and both patients. The spinal cord *D*_2_ was similar for the pCT, rpCT and rpCT with adapted plan for Pat5, while for Pat2 it rose from 42 Gy to 47 Gy and 45 Gy respectively. The adapted plan did not reduce the spared parotid gland *D*_mean_ for both patients. In both cases, the adapted plan on the vCT exhibits similar DVH curves as the original plan on the pCT.

**Fig. 3:**
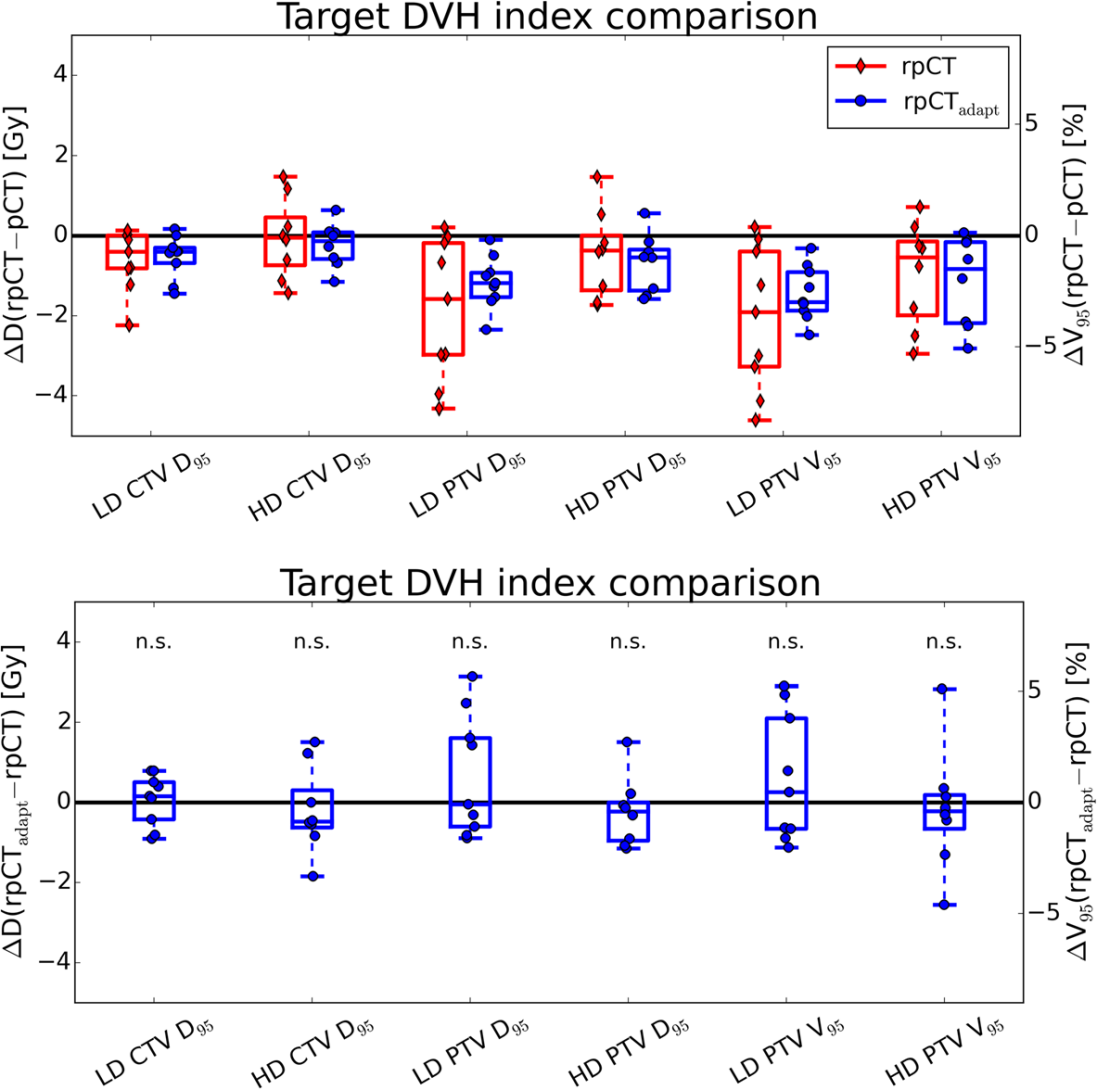
Boxplots of target DVH index (*D*_95_ left vertical axis, *V*_95_ right vertical axis) differences. The original (rpCT) and adapted plans (rpCT_adapt_) on the rpCT are compared to the original plan on the pCT (top panel) and to each other (bottom panel) for the 9 patients investigated in this study. Non-significant differences between original and adapted plan on the rpCT are indicated by ‘n.s.’. All dose values refer to the total dose of the SIB treatment phase for ease of interpretation

**Fig. 4:**
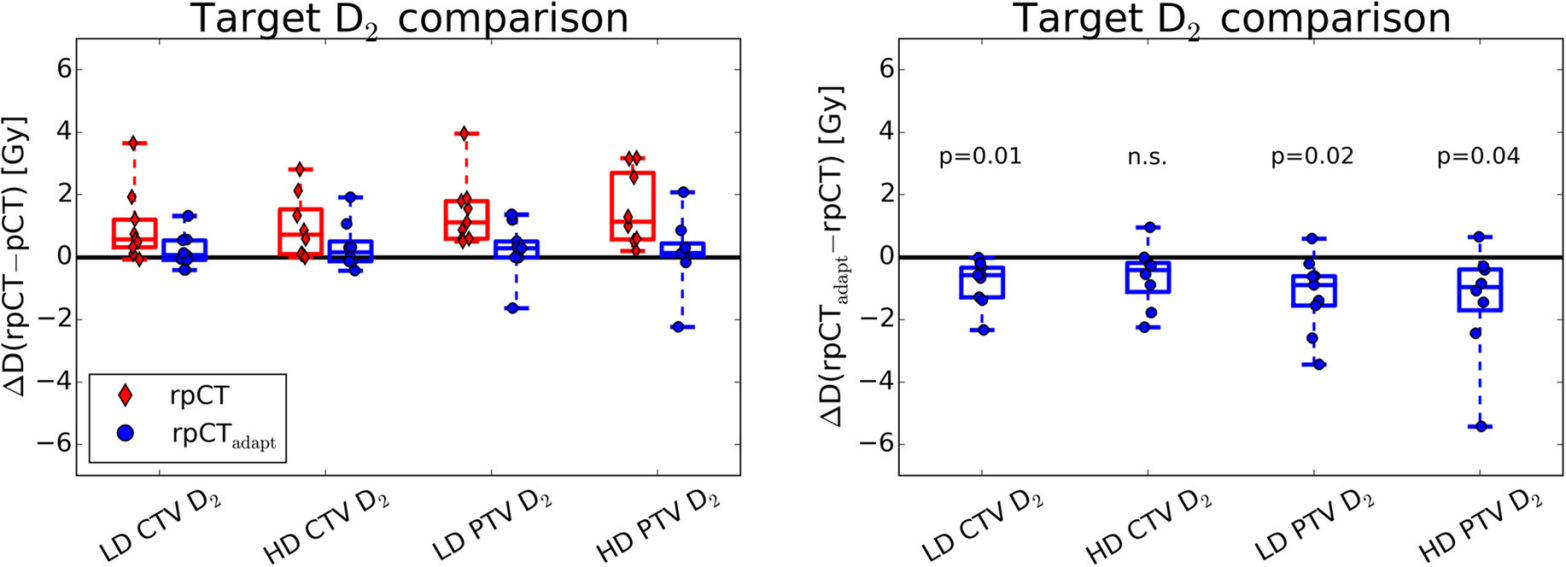
Boxplots of target DVH index (*D*_2_) differences. The original (rpCT) and adapted plans (rpCT_adapt_) on the rpCT are compared to the original plan on the pCT (left panel) and to each other (right panel, including results of the paired Wilcoxon signed-rank test) for the 9 patients investigated in this study. All dose values refer to the total dose of the SIB treatment phase

Figures 3 and 4 present box plots of the distribution of differences of DVH indices for the target volumes of all patients. Absolute differences of the original and adapted plan on the rpCT with respect to the original dose distribution on the pCT are shown (top row in Fig. 3, left in Fig. 4), as well as DVH index differences between original and adapted plan on the rpCT (bottom row in Fig. 3, right in Fig. 4). The Wilcoxon rank-sum test showed that there were no significant differences in the high and low dose CTV and PTV *D*_95_ and *V*_95_ between the original and adapted plan on the rpCT (*p* > 0.05). In terms of *D*_2_, the adapted plans showed improved results for the high and low dose PTVs, as well as for the low dose CTV, with *p* < 0.05. The effect on the high dose CTV was less pronounced (*p* = 0.1, see Fig. 4).

**Fig. 5:**
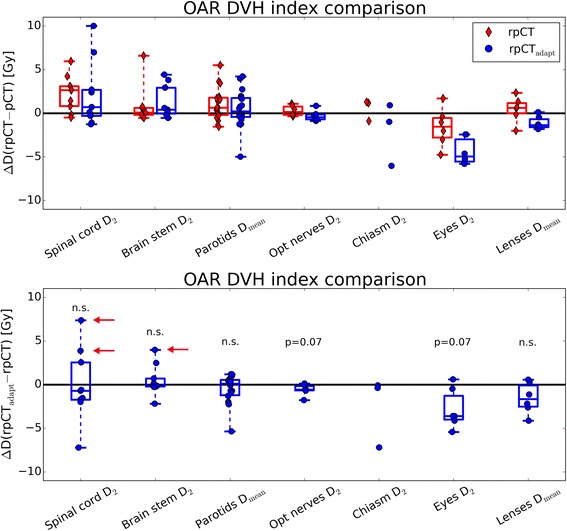
Boxplots of OAR DVH index differences. The original (rpCT) and adapted plans (rpCT_adapt_) on the rpCT are compared to the original plan on the pCT (top panel) and to each other (bottom panel, including results of the paired Wilcoxon signed-rank test) for the 9 patients investigated in this study. Left and right eyes, optical nerves and lenses were grouped together for Pat7-9, left and right parotid glands for all patients. All dose values refer to the total dose of the SIB treatment phase for ease of interpretation. Red arrows indicate the cases with most pronounced OAR dose increase from original to adapted plan (see Discussion section)

Figure 5 shows the data for OARs in similar fashion as Figs. 3 and 4. For OARs, the adapted plan showed no remarkable improvement with respect to the original plan in the spinal cord, brain stem and parotid glands. Differences between the distributions were not significant. However, in two cases, spinal cord *D*_2_ was increased by more than 3 Gy (28 Gy vs 24 Gy and 40 Gy vs 32 Gy) for the adapted plan with respect to the original plan. A considerable brain stem *D*_2_ increase by 4Gy (26 Gy vs 22 Gy) was found in one case (see red arrows in Fig. 5). Still, for all cases the spinal cord and brain stem *D*_2_ were well below the 53 Gy maximum dose objective used for treatment planning. For optical nerves *D*_2_, chiasm *D*_2_, eye *D*_2_, and eye lenses *D*_mean_ we observed a lower dose with rpCT_adapt_ at borderline significance (p in the order of 0.1).

## Discussion

In terms of the high and low dose PTVs and CTVs *D*_95_ and *V*_95_, the adapted plans yielded only minor differences compared to the original plans when evaluated on the rpCT, as observed on Fig. 3. The main improvement from the CBCT-based plan adaptation was the reduction of over-dosage in the target volumes, as indicated by the significantly reduced *D*_2_ values (see Fig. 4). While over-dosage in the GTV may not be of issue, the low dose PTV in H&N cancer patients contains normal tissue where very high doses may not be desired.

**Fig. 6:**
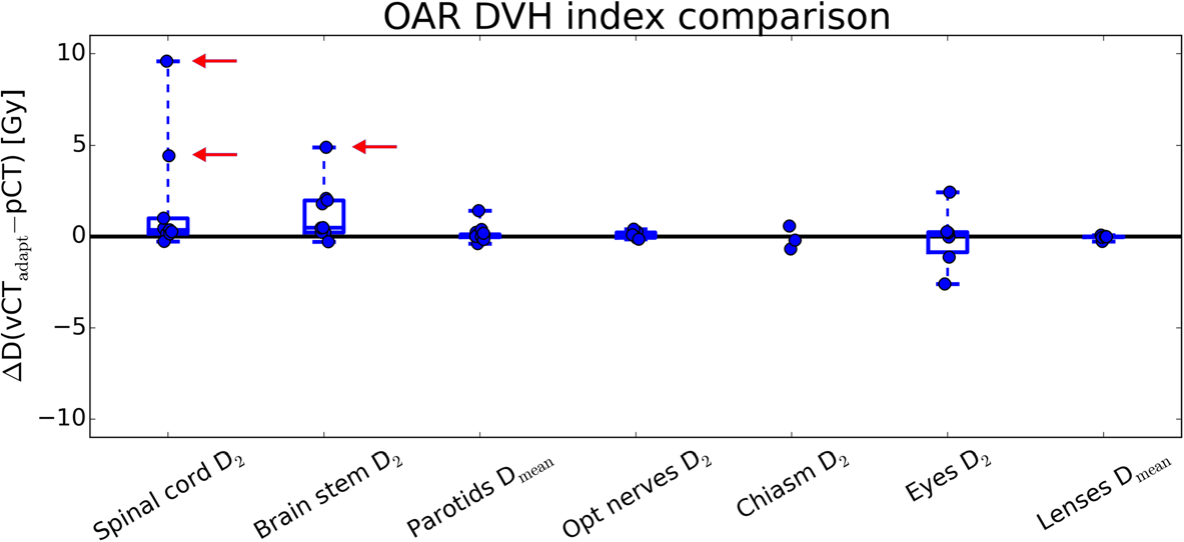
Boxplots of OAR DVH index differences. The vCT optimized plan on the vCT is compared to the original planning scenario (original plan on pCT) for the 9 patients investigated in this study. Red arrows indicate the cases with most pronounced OAR dose increase for the adapted plan. All dose values refer to the total dose of the SIB treatment phase

For OARs, the adapted plans yielded improved results, i.e., lower DVH parameter, for optical nerves, chiasm, eyes and eye lenses with respect to the original plan. For the spinal cord, brain stem and parotid glands, no significant improvement by plan adaptation was found. With respect to the original planning scenario, the adapted plan evaluated on the rpCT shows a higher median OAR dose burden for parotid glands, brain stem and spinal cord (see Fig. 5, top row, blue boxplots). This indicates that the approach investigated here may not be sufficient to ensure optimal sparing of the OARs. However, following optimization of the adapted plan on the vCT, optimization targets for OARs, as well as all target structures, were always met within 1–2 Gy and corresponded to the original plan in terms of DVH parameter, except from the above mentioned three cases with considerably increased spinal cord or brain stem *D*_2_ (see Fig. 2 and Fig. 6). This is probably due to the fact that DVH values were far below the constraints used for plan optimization. In these cases, the dose to the spinal cord or brainstem, respectively, could easily be reduced to a level similar to the original planning scenario by using tighter constraints on the OAR dose during plan adaptation. Dose to other OARs or target structures were not compromised (see Additional file 1: Figure S1 for an exemplary case). This issue might be solved by automatic constraint adaptation as proposed in Breedveld et al. [30].

Remaining differences of the adapted plan recalculated on the rpCT with respect to the vCT, e.g., in terms of the parotid glands *D*_mean_ and the low dose PTV *D*_95_ and *V*_95_, can be due to several reasons: the IMPT plans may not be robust and slight differences in positioning, e.g., different neck tilts, between rpCT and vCT may be sufficient to negate optimal OAR sparing. Beyond, the DIR contours on the vCT could be inaccurate or there could be variations in physician contouring on the rpCT. The pCT, CBCT and rpCT alignments were performed by focusing on a region of interest covering the 1^st^ to 6^th^ vertebra and manual adjustment of the registration yielded corrections of less than 1 voxel. However, by aligning the images using the spine there may be misalignment in other regions of the larger, low dose PTV. For a single patient we also employed a physician delineated vCT to generate the adapted plans and no marked differences were observed between that plan and the one optimized using the DIR delineation. Despite a comprehensive evaluation of the vCT approach by our group [22, 23], we cannot finally conclude on the basis of our patient cohort to what extent differences between vCT and rpCT (using the adapted plan) are related to the effects described above or to non-contour-related inaccuracies of the vCT. To do so, a cohort of IMPT patients with little anatomical changes between repeated diagnostic CT imaging might be investigated to quantify the changes in DVH parameters solely due to re-positioning and re-contouring. In this respect, systematically employing automatic delineation tools may help reduce variability in delineation [31]. To preserve the DVH parameters obtained when optimizing the plan using the vCT it may also be necessary to employ robust optimization, or to use either different or automatically selected beam angles. A robust plan may provide worse parotid sparing initially but may mitigate the increased parotid dose observed on the rpCT.

The adapted plans were generated using a workflow where no human interaction was required in terms of contouring or optimization function adjustment. The procedure could thus be automated and started following the acquisition of the CBCT scan. Online adaptation is not realistic with this procedure given the computational time required for DIR, optimization and dose calculation (total of about 1 h) and the method would thus serve to update the plan for the next fraction. This is the setting we investigated by making use of rpCTs acquired at a different time than the CBCT. We can assume that the anatomical and positioning differences between the rpCT acquisition and CBCT acquisition simulated the differences between two fractions. One key aspect of the vCT approach is that the patient anatomy and position correspond to the situation at the treatment couch, as opposed to the rpCT scan. Development of graphics processor unit based DIR and dose calculation may eventually allow the procedures described here to be performed online. If the vCT approach is not used to automatically adapt the treatment plan, it may still be used to evaluate the need for adaptation by monitoring DVH parameters, dose distributions directly or changes in the water equivalent thickness along beam paths.

## Conclusion

We have established an offline automatic procedure to generate an adapted IMPT plan on CBCT images. When evaluating the adapted plan on a physician delineated control rpCT we observed reduced over-dosage in the high and low dose PTV. OAR sparing was partially improved by the procedure, mainly for the optical system, but might demand for tighter constraints during plan adaptation. Despite potential limitations in the accuracy of the vCT approach, an improved quality of the adapted plans could be achieved. The implementation of the procedure in a clinical workflow would require evaluation of the adapted plans.

## Additional file

Additional file 1: Figure S1. DVH curves for Pat3. The adapted plan (calculated on the vCT) is compared to a re-optimized plan with a tighter constraint of 22 Gy for the maximum dose in the spinal cord. The original adapted plan is labelled vCT on the figure, while the plan with tighter constraints is labelled vCT OAR. **Table S1.** Patient target structure volumes from the pCT and rpCT. All volumes in cm^3^. Patient neck volume changes were computed using the body contour in the neck region, excluding slices below the shoulders and above the jaw. This was not computed for patients with nasal cavity lesions. (PDF 279 kb)

## Abbreviations

ART: adaptive radiotherapy
CBCT: cone beam computed tomography
CT: computed tomography
CTV: clinical target volume
DIR: deformable image registration
DVH: dose/volume histogram
GTV: gross tumor volume
H&N: head and neck
HD: high dose
IMPT: intensity modulated proton therapy
IMRT: intensity modulated radiation therapy
L: left
LD: low dose
OAR: organ at risk
pCT: planning CT
PRV: planning risk volume
PTV: planning target volume
ROI: region of interest
rpCT: replanning CT
SIB: simultaneous integrated boost
TPS: treatment planning system
vCT: virtual CT
