# Supplementary material for: Feasibility of automated proton therapy plan adaptation for head and neck tumors using cone beam CT images
Source: Radiat Oncol. 2016 Apr 30;11:64. doi: 10.1186/s13014-016-0641-7 (PMC4851791; doi:10.1186/s13014-016-0641-7)
Supplement: Additional file 1: Figure S1. — DVH curves for Pat3. The adapted plan (calculated on the vCT) is compared to a re-optimized plan with a tighter constraint of 22 Gy for the maximum dose in the spinal cord. The original adapted plan is labelled vCT on the figure, while the plan with tighter constraints is labelled vCT OAR. Table S1. Patient target structure volumes from the pCT and rpCT. All volumes in cm3. Patient neck volume changes were computed using the body contour in the neck region, excluding slices below the shoulders and above the jaw. This was not computed for patients with nasal cavity lesions. (PDF 279 kb) [file 13014_2016_641_MOESM1_ESM.pdf]

Additional file 1

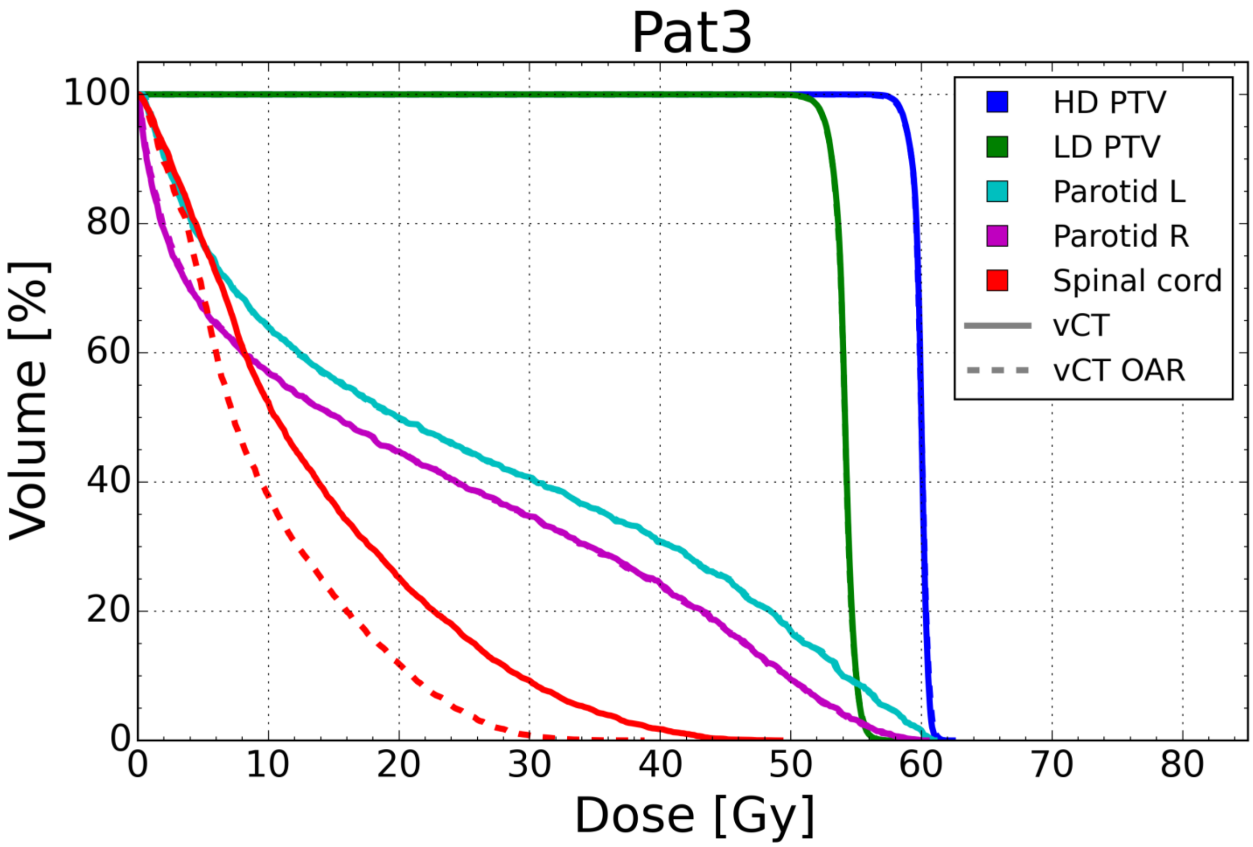

Figure S1. DVH curves for Pat3. The adapted plan (calculated on the vCT) is compared to a re-optimized plan with a tighter constraint of 22 Gy for the maximum dose in the spinal cord. The original adapted plan is labelled vCT on the figure, while the plan with tighter constraints is labelled vCT OAR.

Table S1. Patient target structure volumes from the pCT and rpCT. All volumes in cm^3. Patient neck volume changes were computed using the body contour in the neck region, excluding slices below the shoulders and above the jaw. This was not computed for patients with nasal cavity lesions.

Patient identifier	LD CTV		LD PTV		HD CTV		HD PTV		Neck volume change rpCT vs pCT
	pCT	rpCT	pCT	rpCT	pCT	rpCT	pCT	rpCT	(%)
Pat1	179	180	418	418	-	-	-	-	0
Pat2	564	491	1087	983	49	34	127	102	-8
Pat3	365	359	772	762	157	158	345	352	-4
Pat4	637	666	1129	1114	206	211	408	407	1
Pat5	985	992	1783	1769	349	354	790	797	-5
Pat6	535	550	996	998	193	193	386	390	-3
Pat7	300	293	516	510	-	-	174	173	-
Pat8	637	640	987	980	70	70	129	129	-
Pat9	888	913	1639	1655	118	113	243	238	-
